# Coagulopathy and Fibrinolytic Pathophysiology in COVID-19 and SARS-CoV-2 Vaccination

**DOI:** 10.3390/ijms23063338

**Published:** 2022-03-19

**Authors:** Shinya Yamada, Hidesaku Asakura

**Affiliations:** Department of Hematology, Kanazawa University Hospital, Takaramachi 13-1, Kanazawa 920-8640, Ishikawa, Japan; abacus3shinya@gmail.com

**Keywords:** COVID-19, SARS-CoV-2 vaccine, coagulopathy, fibrinolysis, enhanced-fibrinolytic-type DIC, nafamostat

## Abstract

Coronavirus Disease 2019 (COVID-19) is caused by severe acute respiratory syndrome coronavirus 2 (SARS-CoV-2) and is frequently complicated by thrombosis. In some cases of severe COVID-19, fibrinolysis may be markedly enhanced within a few days, resulting in fatal bleeding. In the treatment of COVID-19, attention should be paid to both coagulation activation and fibrinolytic activation. Various thromboses are known to occur after vaccination with SARS-CoV-2 vaccines. Vaccine-induced immune thrombotic thrombocytopenia (VITT) can occur after adenovirus-vectored vaccination, and is characterized by the detection of anti-platelet factor 4 antibodies by enzyme-linked immunosorbent assay and thrombosis in unusual locations such as cerebral venous sinuses and visceral veins. Treatment comprises high-dose immunoglobulin, argatroban, and fondaparinux. Some VITT cases show marked decreases in fibrinogen and platelets and marked increases in D-dimer, suggesting the presence of enhanced-fibrinolytic-type disseminated intravascular coagulation with a high risk of bleeding. In the treatment of VITT, evaluation of both coagulation activation and fibrinolytic activation is important, adjusting treatments accordingly to improve outcomes.

## 1. Introduction

The novel coronavirus disease 2019 (COVID-19), which was first identified in Wuhan in December 2019, is caused by severe acute respiratory syndrome coronavirus 2 (SARS-CoV-2). As of the end of January 2022, approximately two years had passed since the beginning of the COVID-19 pandemic, with the number of infected people worldwide exceeding 3.5 billion and the number of deaths exceeding 550 million at that time. COVID-19 is often complicated by coagulopathy and thrombosis. Though immobility of severely ill patients needs to be considered [1], the incidence of thrombosis appears high among severe COVID-19 cases [2,3].

SARS-CoV-2 vaccines have been developed by many countries to prevent the spread of the infection and reduce the severity of COVID-19. However, it is known that vaccination rarely causes coagulopathy [4].

**Figure 1:**
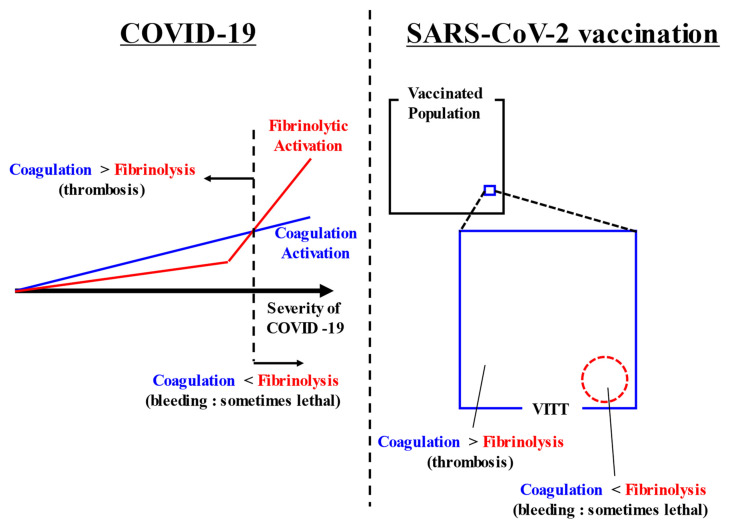
Summarizing figure. COVID-19 causes coagulation activation depending on its severity. In addition, some cases of severe COVID-19 have markedly increased fibrinolysis. Thrombosis appears as the main symptom when coagulation activation exceeds fibrinolytic activation. Conversely, bleeding appears as the main symptom when fibrinolytic activation exceeds coagulation activation. After SARS-CoV-2 vaccination, VITT rarely occurs. In most cases, coagulation activation exceeds fibrinolytic activation and thrombosis occurs, but in some cases, bleeding appears when fibrinolytic activation exceeds coagulation activation. Abbreviations: COVID-19, coronavirus disease 2019; SARS-CoV-2, severe acute respiratory syndrome coronavirus 2; VITT, vaccine-induced immune thrombotic thrombocytopenia.

In both COVID-19-associated coagulopathy and SARS-CoV-2 vaccination-associated coagulopathy, attention to both thrombosis and bleeding is required. While much attention has been given to thrombosis and coagulation activation, almost never have reports discussed bleeding and fibrinolysis. We would therefore like to highlight and discuss these relatively neglected factors. A summarizing figure is shown in (Figure 1).

## 2. COVID-19-Associated Coagulopathy

In COVID-19-associated coagulopathy, concentrations of D-dimer, von Willebrand factor (VWF), and interleukin (IL)-6 are elevated with increasing severity of the disease, and the incidence of thrombosis is higher in severe cases. Two theories have been proposed for the mechanisms underlying COVID-19-associated coagulopathy [5].

The first is that SARS-CoV-2 infects endothelial cells and causes thrombosis with vascular inflammation [6]. Electron microscopy [7,8], immunohistochemistry [9,10,11], and in situ hybridization [9,10,12] have shown SARS-CoV-2 itself, viral particles, and coronavirus-like particles in endothelial cells. These reports support the theory that SARS-CoV-2 directly infects endothelial cells and impairs their antithrombogenic properties. Damage to endothelial cells by viral infection could readily be understood as a cause of thrombosis. However, the mechanism by which SARS-CoV-2 enters vascular endothelial cells through binding to the angiotensin-converting enzyme 2 (ACE2) receptor of such cells [13] has not been demonstrated in endothelial cells in vivo. Furthermore, the structures in endothelial cells interpreted as representing SARS-CoV-2 and coronavirus-like particles have been suggested to be misinterpretations of coated vesicles and multivesicular bodies [14,15].

A second theory is that SARS-CoV-2 does not directly infect endothelial cells, but rather, that cytokine storms as excessive immune responses are responsible for thrombosis. Although ACE2 receptors, as the host receptor for SARS-CoV-2, have been thought to be highly expressed in vascular endothelial cells [16], endothelial cells may actually not express ACE2 receptors [17,18] or may only express ACE2 receptors at very low levels [19]. Another study found that cultured endothelial cells were resistant to SARS-CoV-2 infection [20]. However, infection of alveolar epithelial cells and alveolar macrophages with SARS-CoV-2 results in these cells producing markedly elevated levels of cytokines or chemokines such as IL-6, IL-8, tumor necrosis factor (TNF)-α, and C-X-C motif chemokine ligand 8 (CXCL8), resulting in a cytokine storm [21,22,23]. Cytokine storms are thought to lead to platelet activation, coagulation activation, endothelial injury, and a consequent decrease in endothelial antithrombogenic activity and increase in prothrombogenic activity [5]. Furthermore, the SARS-CoV-2 spike protein alone has been reported to stimulate endothelial activation [24], resulting in cytokine release and complement activation [25].

Considering that direct entry of SARS-CoV-2 into endothelial cells has not been confirmed and that entry of SARS-CoV-2 is not necessarily required for endothelial cell damage [26], the latter theory may be more relevant at present, but clarification of these issues is expected in the near future.

### 2.1. COVID-19 and Physical Findings

Urticarial rash reportedly accounts for more than 10% of COVID-19-related skin findings [27,28]. Chilblain-like acral patterns [29,30,31,32] are not rare among young adults and children and have gained attention in social media. Pathologically, epidermal necrotic keratinocytes, dermal edema, endotheliitis, and microthrombi have been identified [31,33]. Livedo reticularis [34,35,36,37] is found in approximately 5% of COVID-19-associated skin lesions [28], reflecting impaired skin circulation, and histopathological examination shows inflammatory micro-thrombotic vasculopathy [38]. Attention to skin findings may facilitate early diagnosis of COVID-19.

Apart from skin findings, an interesting report stated that in the early stage of COVID-19, oral ulcer lesions were found in 65.9% of cases and histopathological examination showed thrombosis in small and middle-sized vessels [39]. If COVID-19 is suspected, intraoral findings should also be noted.

Cases have also been reported in which COVID-19 was diagnosed subsequent to deep vein thrombosis or abdominal pain (visceral vein thrombosis), despite the absence of any respiratory symptoms [40]. Many reports have also described lower limb ischemia from intra-aortic thrombi [41,42,43,44].

Thrombosis caused by COVID-19 is most commonly identified in the lungs, but can occur in various parts of the body, with associated physical findings. COVID-19 should therefore be included among the differential diagnoses when physical findings suggest thrombosis, particularly during the current pandemic.

### 2.2. D-Dimer and Prognostic Factors for Poor Clinical Outcome in COVID-19

Elevated levels of D-dimer have been reported as a prognostic factor for poor clinical outcome in COVID-19 [45,46,47,48,49]. High D-dimer levels are associated with COVID-19 severity [50,51], death [47,52], and the development of venous thromboembolism [53,54].

High D-dimer levels, however, are not only reflective of thrombosis; the progressive lung tissue damage associated with COVID-19 may also lead to the formation of fibrin in the alveoli and lung parenchyma, the degradation products of which subsequently enter the bloodstream [55,56,57,58]. This may be another reason why elevated D-dimer levels are closely associated with poor prognosis, as high D-dimer levels can reflect either thrombosis or lung tissue damage.

In addition to D-dimer, other markers predicting poor prognosis include lymphocytopenia [59,60], thrombocytopenia [59,60], elevated C-reactive protein [61,62], elevated IL-6 [60,61,62], elevated lactate dehydrogenase (LDH) [63], elevated ferritin [60], sphingolipid profile [64], increases in soluble tumor necrosis factor or its receptor, or an increase in a disintegrin and metalloproteinase 17 (ADAM17) [65]. Other risk factors for severe disease and death include advanced age [48], obesity [66], vitamin D deficiency [67], zinc deficiency [68], and coexisting cardiovascular disease [69]. Combinations of these factors may thus contribute to more accurate assessment of prognosis. In addition, corrections of abnormalities in some of these factors in advance are expected to contribute to prevention of SAR-CoV-2 infection or COVID-19 exacerbation.

In addition to high D-dimer levels, other clotting abnormalities that predict poor prognosis include a slow decline in D-dimer after anticoagulation [70]; D-dimer should be tracked over time, not simply at COVID-19 diagnosis, and adjusting the dose of anticoagulation based on such tracking may be important.

Prolonged prothrombin time (PT) [71], decrease in antithrombin activity (AT) [72], and decrease in a disintegrin-like metalloprotease with thromboplastin type 1 motif 13 (ADAMTS13) activity [73] at the time of COVID-19 diagnosis are also predictors of poor prognosis. A decrease in ADAMTS13 activity may be a result of the consumption of ADAMTS13 during the cleavage of VWF [74], which is overexpressed following damage to the vascular endothelium. In other words, elevated VWF and decreased ADAMTS13 may represent markers of a thrombotic tendency, along with D-dimer. These markers reportedly reflect strong inflammation and vascular endothelial damage [75].

Platelet-related markers such as the presence of platelet aggregates [76], platelet activation (high mean platelet volume (MPV) and high immature platelet fraction (IPF)) [77], and high immature platelet count (IPC) [78] have also recently been considered as predictors of poor prognosis. As platelets mature, their volume and MPV both decrease. Meanwhile, immature platelets show a larger volume and higher activity. Both these markers indicate increased platelet consumption and production. MPV has an advantage, in that the data exist for all patients who have had a hemogram. However, MPV is disadvantageous in that the value cannot be used when platelet counts are very low; in such cases, IPF should be used. IPF is not currently available at all medical institutions, but is a useful marker for differentiating conditions in which the platelet count is low

### 2.3. COVID-19 and Thrombotic/Hemorrhagic Disease

In COVID-19, low platelet count [59,60] and platelet hyperfunction [76,77,78] are important predictors of poor prognosis. Conditions with marked platelet activation and high consumption are thought to be associated with poor prognosis. Diseases and conditions that can cause thrombocytopenia in COVID-19 are summarized in Table 1. Some of these conditions can be cured by early diagnosis and treatment, so differentiating between etiologies is necessary when platelet counts are decreased.

Thrombosis occurring in COVID-19 can be classified as either “macro-thrombosis” or “micro-thrombosis”, according to size. These two types of thrombosis are thought to differ not only in size, but also in etiology and response to treatment.

#### 2.3.1. Macro-Thrombosis in COVID-19

Macro-thrombosis is an arterial or venous thrombosis of varying size that can be diagnosed from imaging studies such as contrast-enhanced CT or ultrasonography. Among 184 COVID-19 patients who entered an intensive care unit (ICU), 40% had macro-thrombosis [82]. However, the actual frequency of macro-thrombosis in severe COVID-19 is not known, due to the difficulty of performing adequate contrast-enhanced CT examinations in patients with severe disease admitted to the ICU [83]. The actual frequency of macro-thrombosis in severe COVID-19 may thus be even higher.

#### 2.3.2. Micro-Thrombosis in COVID-19

Micro-thrombi are not detectable on imaging studies, and most micro-thrombi are pathologically confirmed on autopsy [84,85,86]. Although COVID-19 causes a systemic tendency toward thrombosis, the involvement of the lung in this is overwhelmingly common, with multiple thrombi in the pulmonary arteriovenous system [84,85,86]. These thrombi contain both fibrin and platelet components [86], and an autopsy report of the site of SARS-CoV-2 revealed an overwhelming amount of SARS-CoV-2 in the lungs [11]. This may be because the ACE2 receptor, the host-side receptor for SARS-CoV-2, is highly expressed in alveolar epithelial type II cells [16]. In the lungs, endothelial damage with macrophage activation, complement activation, tissue factor upregulation, and loss of thrombomodulin [87] may proceed, resulting in the formation of massive thrombi [57]. As mentioned above, the origin of the elevated plasma D-dimer in patients with COVID-19 is thought to reflect not only the origin of the thrombi in circulating blood but also the degradation of fibrin clots formed in the alveoli or lung parenchyma and entry of the byproducts of this degradation into the circulation. Since these sites of fibrin formation are most common in the lung, pulmonary intravascular coagulation (PIC), rather than disseminated intravascular coagulation (DIC), has been suggested as the more appropriate term for coagulation abnormalities in COVID-19 [88,89,90]. Since micro-thrombosis involves various factors other than platelet and coagulation activation, such as neutrophil extracellular traps [91,92] and vascular endotheliitis [7], the efficacy of antiplatelet and anticoagulant drugs against micro-thrombosis may be even less than that against macro-thrombosis

### 2.4. Dynamic Changes in Coagulation/Fibrinolysis in COVID-19

The incidence of DIC in COVID-19 was 0.6% in patients who survived, compared to 71.4% in patients who died [45]. In other words, DIC is more likely to occur only in severe cases. Survival in cases of DIC also appears difficult.

In general, DIC due to severe infection or sepsis is considered to represent suppressed-fibrinolytic-type DIC [93]. In suppressed-fibrinolytic-type DIC, the thrombin–antithrombin complex (TAT), representing a marker of coagulation activation, is markedly increased, whereas the plasmin-α2 plasmin inhibitor complex (PIC), as a marker of fibrinolytic activation, is only mildly elevated. In other words, clots are formed but do not lyse sufficiently. Ischemic organ damage due to multiple micro-thrombi is thus easily observed. Owing to the lack of thrombus dissolution, levels of fibrin/fibrinogen degradation products (FDP) and D-dimer are only mildly elevated [93]. Ergo, what is the situation in COVID-19?

Important detailed and longitudinal follow-up reports of changes to coagulation/fibrinolysis markers in COVID-19 have been published in China [45] and Japan [94].

In a report by Tang et al. from China [45], PT, activated partial thromboplastin time (APTT), fibrinogen, FDP, D-dimer, and AT were analyzed on days 1, 4, 7, 10, and 14 after admission for 183 COVID-19 patients (162 survivors, 21 non-survivors). PT was significantly prolonged and FDP and D-dimer were significantly increased among non-survivors. Fibrinogen increased to more than 400 mg/dL (normal range: 200–400 mg/dL) on day 7 in both survivors and non-survivors, but fibrinogen decreased to about 100 mg/dL on day 10 only in non-survivors. In addition, only fatal cases showed a marked increase in FDP and an increase in the FDP/D-dimer ratio (reflecting the discrepancy between FDP and D-dimer) during the 3 days from day 7 to day 10. Patients were thought to present with suppressed-fibrinolytic-type DIC on admission, suddenly changing to enhanced-fibrinolytic-type DIC between days 7 and 10 [95].

A report by Ishikura et al. from Japan [94] described the detailed clinical course, PT, APTT, fibrinogen, FDP, D-dimer, and AT, along with TAT, PIC, and plasminogen activator inhibitor-1 (PAI-1) in six patients (four survivors, two non-survivors) admitted to the ICU. In addition to PT, APTT, fibrinogen, FDP, D-dimer, and AT, the coagulation activation marker TAT, the fibrinolytic activation marker PIC, and the fibrinolytic inhibition marker PAI-1 were tracked daily. Similar to the report by Tang et al., Ishikura et al. found not only a marked decrease in fibrinogen, a marked increase in FDP, and an increase in FDP/D-dimer ratio among some patients after seven days in the ICU, but also a marked increase in the fibrinolytic activator marker PIC. This further supported the existence of a dynamic change from fibrinolytic suppression to fibrinolytic enhancement in just one day. In particular, the presence of PIC > 40 μg/mL (reference: ≤0.8 μg/mL) is striking. Even in aortic aneurysms and vascular malformations, which are among the most common diseases causing enhanced-fibrinolytic-type DIC, PIC is approximately 10 μg/mL [96,97,98]. PIC is also around 10 μg/mL in acute promyelocytic leukemia, in which fatal bleeding can arise if therapeutic intervention is delayed [93]. Meanwhile, in addition to the findings from Ishikura et al., a marked increase in PIC to >10 μg/mL in COVID-19 patients with confirmed PIC has been reported [99]. Such a large increase in PIC is rarely seen in patients other than those with COVID-19 [100]. In addition, Ishikura et al. [94] reported cerebral hemorrhage in patients with markedly increased PIC, suggesting that fibrinolytic activation in COVID-19 is associated with hemorrhage.

**Figure 2:**
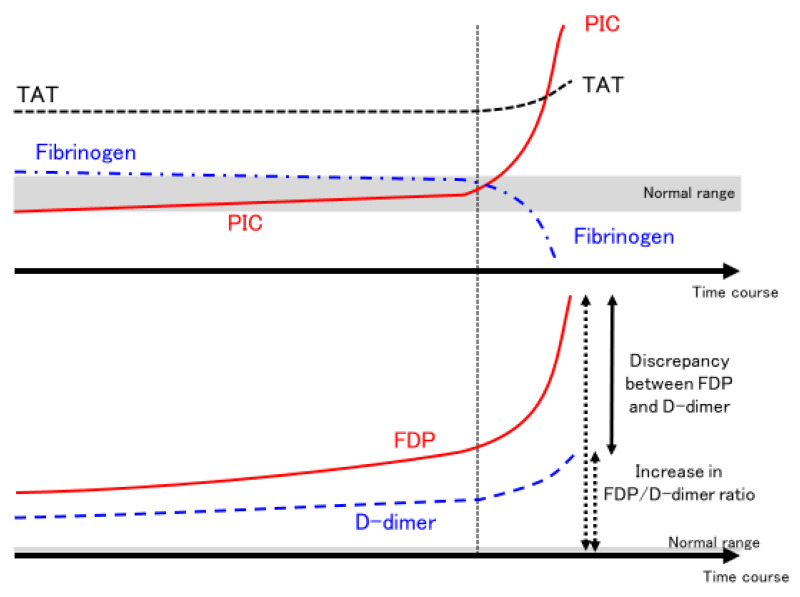
Dynamic changes in fibrinolytic pathophysiology in some severe COVID-19 cases. The time course of coagulation markers in COVID-19 patients with sudden enhancement of fibrinolytic activation and severe bleeding are shown. In some severe COVID-19 patients, PIC suddenly increases (PIC surge) at a certain point (around 7–10 days after admission to the ICU, as indicated by the vertical dotted line). At the same time, fibrinogen decreases significantly, but the degree of increase in TAT does not change significantly (upper panel). Meanwhile, FDP increases significantly, while D-dimer increases only mildly. Both solid and dotted arrows indicate the FDP/D-dimer ratio (lower panel). As PIC increases and fibrinogen decreases, discrepancy between FDP and D-dimer or the FDP/D-dimer ratio increases. These changes in coagulation markers are suggestive of enhanced-fibrinolytic-type DIC. Abbreviations: COVID-19, coronavirus disease 2019; ICU, intensive care unit; PIC, plasmin–α_2_ plasmin inhibitor complex; TAT, thrombin–antithrombin complex; FDP, fibrin/fibrinogen degradation products; DIC, disseminated intravascular coagulation.

Whether elevated PIC is associated with fatal hemorrhage and death remains to be systematically investigated, but several autopsy cases have shown prominent hemorrhage as well as thrombosis [84]. This suggests that in a subset of severe COVID-19 cases, fibrinolysis may suddenly become markedly activated, resulting in hemorrhage (Figure 2) [95,101]. For patients with moderate COVID-19, the use of therapeutic doses of heparin contributed to an increase in survival to hospital discharge and a decrease in embolic events [102]. However, for patients with severe COVID-19, heparin was not only ineffective, but also did not improve the probability of survival to hospital discharge and may have increased major bleeding [103]. This may be due to the possibility that therapeutic doses of heparin induced major bleeding in enhanced-fibrinolytic-type DIC, which is thought to be present in some critically severe patients, in addition to the possibility that heparin was not fully effective in patients with severe COVID-19. This is an issue for further investigation.

In COVID-19 clinical practice, in addition to the global markers PT, APTT, fibrinogen, FDP, D-dimer, and AT, the coagulation activation marker TAT, the fibrinolysis activation marker PIC, plasminogen, and the alpha2 plasmin inhibitor (alpha2 PI) (α2PI < 50% increases the risk of bleeding) should also be measured [93]. However, in many medical facilities, tests such as TAT and PIC must be outsourced, and checking the results on the same day and adjusting the treatment plan accordingly is therefore difficult. In such cases, a decrease in fibrinogen, a marked increase in FDP, a moderate increase in D-dimer, and an increase in the FDP/D-dimer ratio should be considered as indicators of significant fibrinolytic activation, and the test results should be utilized to inform adjustments to treatment plans on the same day [93,95,101,104]. In addition, medical facilities that treat a large number of patients with severe COVID-19 should work to obtain the capability to measure TAT and PIC at their own facilities.

### 2.5. Treatment of COVID-19-Associated Coagulopathy

In general, thrombosis is a common complication of COVID-19, with a higher incidence of thrombosis in severe cases [2,3]. However, the reported incidence of thrombosis should be interpreted with caution. In other words, whether contrast-enhanced computed tomography (CT) or venous echocardiography are actively performed in patients with COVID-19 needs to be considered. If contrast-enhanced CT is performed infrequently, thrombosis may frequently be missed [83]. In addition, assuming the presence of micro-thrombosis that cannot be detected by contrast-enhanced CT, the number of thromboses may be much higher than reported.

On the other hand, advanced fibrinolytic activation and severe bleeding symptoms are seen in some severe cases. In addition to the transition to enhanced-fibrinolytic-type DIC, other causes of bleeding in severe COVID-19 include side effects of anticoagulants used to prevent or treat thrombosis, vascular fragility associated with endotheliitis, acquired von Willebrand syndrome under extracorporeal membrane oxygenation (ECMO), and low platelet counts. The causes of bleeding in COVID-19 are summarized in Table 2 [95,99,101,105,106]. Concern has been raised that bleeding seen during anticoagulation for COVID-19 might easily be considered a side effect of anticoagulation, whereas bleeding in COVID-19 can have many causes, and appropriate differentiation is important.

When bleeding emerges in severe COVID-19 patients, measurement of PT, APTT, fibrinogen, FDP, D-dimer, AT, TAT, PIC, plasminogen, and αPI2 represents the first step toward appropriate differentiation. If the patient is under ECMO, VWF antigen, VWF activity, factor VIII activity, and VWF multimer analysis should be performed to differentiate acquired von Willebrand syndrome [107]. In addition, although blood thrombomodulin levels reportedly reflect vascular endothelial damage [108], thrombomodulin is a renal metabolite and should be assessed with caution, because renal failure will increase blood levels of thrombomodulin regardless of vascular endothelial damage.

#### 2.5.1. Antiplatelet Agents

As mentioned above, values such as MPV and platelet distribution width are elevated in COVID-19 patients, possibly reflecting platelet activation [77,109], and the use of aspirin for at least one week prior to admission significantly reduced the induction of ventilation [110]. The use of antiplatelet agents reduced mortality and ventilator duration [111], decreased in-hospital mortality [112], and slightly increased the proportion of patients discharged alive within 28 days with the use of aspirin among hospitalized patients [113]. On the other hand, some reports have found that antiplatelet therapy was ineffective against COVID-19 [114,115,116].

The reasons for such opposing conclusions regarding antiplatelet therapy may lie in differences of patient populations, duration of antiplatelet therapy, and combined anticoagulant therapy. In addition, patients on aspirin show more risk factors for COVID-19 aggravation such as aging, coronary artery disease, and diabetes [47], warranting careful evaluation.

#### 2.5.2. Heparin Therapy

Heparin exerts anti-inflammatory effects by binding to complement [117,118] and cytokines [119,120], and antiviral effects by inhibiting host cell–virus interactions [121,122,123]. As mentioned above, heparin therapy is effective in cases of mild to moderate COVID-19 [102], but the antiviral effects of heparin and anticoagulation with heparin alone are considered to be limited in severe cases.

In this regard, the development of regimens with other drugs in combination with heparin to improve the therapeutic effects of anticoagulation and to reduce the side effects of bleeding is expected in the future.

#### 2.5.3. Combination Therapy with Heparin and Nafamostat (Attention to Fibrinolytic Pathophysiology)

If bleeding symptoms associated with elevated PIC occur during anticoagulation for severe COVID-19, the intensity of anticoagulation and the type of anticoagulant should be reconsidered. For example, the dose of anticoagulant could be reduced, or nafamostat, a synthetic serine protease inhibitor with potent antiplasmin effects, could be added to the ongoing anticoagulant therapy (heparin-nafamostat combination therapy) [100,124,125,126,127]. Nafamostat has been used in Japan for acute pancreatitis and DIC for more than 30 years, and Japanese clinicians are familiar with this agent.

Nafamostat holds promise in the treatment of COVID-19 for three reasons.

(1) Anti-thrombin effects

Nafamostat has inhibitory effects on serine proteases, inhibiting coagulation factors such as factors VIIa, IXa, Xa, and IIa (thrombin), all of which are serine proteases [128]. These factors are also inhibited by heparin, but heparin is antithrombin-dependent, whereas nafamostat can inhibit these factors in a heparin-independent manner. In other words, nafamostat is sufficiently effective even in patients with reduced AT activity based on the molecular mechanism of the nafamostat.

(2) Anti-plasmin activity

Cleavage of the S protein of SARS-CoV-2 by plasmin has been reported to increase virulence [129], and nafamostat, which also shows antiplasmin effects, may also have antiviral effects in this regard.

(3) Anti-transmembrane serine protease 2 (TMPRSS2) action

In addition, SARS-CoV-2 completes its entry into host cells when the S protein bound to the ACE2 receptor is degraded by the cell surface enzyme TMPRSS2, but nafamostat inhibits the function of TMPRSS2 and thus acts to block this step [130,131,132,133].

In short, nafamostat appears to be an effective COVID-19 treatment that not only acts against thrombin, but also weakens the virulence of the virus itself and inhibits its entry into host cells such as alveolar epithelial cells. Nafamostat is a promising agent, even as a single agent. Nafamostat alone did not affect the clinical improvement of moderate to severe COVID-19, but in the severe subgroup, nafamostat alone improved clinical improvement [134]. However, nafamostat also has the limitation of being a weak anticoagulant, and thus may be best used in combination with heparin [124]. Attention should also be paid to the side effect of hyperkalemia [135,136] from nafamostat. On the other hand, the near-total absence of bleeding side effects with nafamostat represents a major point in its favor, based on our experience with its use in a large number of DIC cases in Japan.

Anticoagulant therapies that also have anti-inflammatory and antiviral effects, such as heparin and nafamostat, hold promise as treatments for COVID-19. Currently, a number of clinical trials investigating nafamostat for COVID-19 are underway [137].

#### 2.5.4. Direct Oral Anticoagulant

Patients with COVID-19 are often treated with heparin during hospitalization to prevent thrombosis. However, the hypercoagulable state often persists after discharge [138,139,140], and some reports have suggested that transition to a direct oral anticoagulant (DOAC) at discharge may be useful [141].

Some patients may have been taking DOACs prior to the onset of COVID-19 and continue to take DOACs. However, concomitant use of antivirals (lopinavir, ritonavir, or darunavir) and DOACs has been reported to markedly increase DOAC blood levels by a mean of 6.14-fold (1.6–31.6 fold) [142]. It has been reported that administration of rivaloxaban to COVID-19 inpatients with elevated D-dimer showed no clinical improvement and increased bleeding [143]. In addition, no appropriate monitoring index for DOACs has yet been established [144], and attention should be paid to the occurrence of bleeding symptoms when DOACs are combined with antiviral drugs.

#### 2.5.5. Fibrinolytic Treatment

In COVID-19, systemic administration of the fibrinolytic agent tissue plasminogen activator (tPA) has been reported to be both effective [145,146,147] and ineffective [148,149]. High levels of PAI-1 [149] have been identified in ineffective cases, suggesting that different patient backgrounds may have led to these widely divergent results, since tPA is not sufficiently effective when PAI-1 is high.

We believe that systemic fibrinolytic therapy in COVID-19 is dangerous to the point of contraindication. Particularly in severe cases of COVID-19, fibrinolysis becomes highly activated in just one to three days [94,95]. We are concerned that fibrinolysis may cause fatal bleeding in such patients.

However, inhalation of fibrinolytic agents such as tPA and plasminogen [150,151] can reduce the risk of bleeding much more than systemic administration and may improve acute respiratory distress syndrome (ARDS) by dissolving fibrin clots in and near the alveolar spaces [152]. Inhaled heparin has also been shown to be effective in the treatment of COVID-19 patients [153,154]. Some problems with inhaled heparin therapy still need to be addressed, such as individual differences in blood transfer and the possibility of exposure of health care providers to droplet infection.

#### 2.5.6. Treatments to Avoid

(a) Warfarin

Even under adequate warfarin control, reports have described cases of elevated D-dimer [155] and pulmonary thromboembolism [156]. The lack of anti-inflammatory and antiviral effects of warfarin compared with heparin and nafamostat may explain the inferior therapeutic efficacy. Moreover, control of warfarin is likely to be greatly disturbed by COVID-19 and its treatment [157,158]. In addition, when COVID-19 is complicated by DIC, warfarin use may potentially exacerbate the DIC [96], so frequent monitoring is recommended when warfarin is needed for patients with COVID-19.

If a patient on warfarin develops COVID-19, consideration should be given to switching the patient to a DOAC for outpatient treatment or heparin for inpatient treatment [96].

(b) Tranexamic acid

The addition of tranexamic acid, an antifibrinolytic agent, to anticoagulation is one method of treatment [100,159,160]. This is because the increased pathogenicity of SARS-CoV-2 by S-protein cleavage can be inhibited via the antiplasmin effect of tranexamic acid [129]. However, given the high degree of coagulation activation in COVID-19, the use of tranexamic acid, even in combination with anticoagulant therapy, may exacerbate thrombosis [161] and should not be used lightly.

## 3. SARS-CoV-2 Vaccination-Associated Coagulopathy

### 3.1. Safety of Vaccination in Persons with Coagulation Abnormalities

Safe administration of vaccines to patients with coagulation abnormalities involves two considerations: first, whether the physical invasion associated with vaccination will cause bleeding in patients with bleeding tendencies or hemorrhagic diseases; and second, whether patients with hemorrhagic or thrombotic diseases will experience exacerbation or flare-up of the underlying disease.

#### 3.1.1. Preventing Bleeding Due to Vaccination in Patients with Coagulation Abnormalities

Vaccination of patients on antiplatelet and anticoagulant medications (warfarin and DOAC) is certainly possible. However, adequate compression of the vaccination site should be performed after vaccination [162]. If the PT-INR is above the therapeutic range, vaccination should be postponed until the PT-INR is back within the therapeutic range [161], and in DOAC-treated patients, vaccination is best avoided when blood levels of DOACs are high.

Vaccination after prophylaxis treatment is recommended in patients with hemophilia who are receiving regular prophylaxis treatment and in patients with severe von Willebrand disease.

Thrombocytopenia/dysfunctional platelets do not interfere with vaccination, but adequate compression of the vaccination site after vaccination is advisable.

In chronic DIC (e.g., cases with aortic aneurysms and vascular malformations), the invasiveness of vaccination may cause severe bleeding and should be discussed with the attending physician [128].

#### 3.1.2. Vaccination and Thrombotic/Hemorrhagic Disease Exacerbations and Relapses

In patients with immune thrombocytopenia (ITP), the relapse of ITP with vaccination represents a matter of concern. In fact, vaccination of patients with ITP resulted in a ≧20% decrease in platelet count from baseline in about half of patients [163]. However, no reports have suggested that vaccination should be withdrawn in patients with ITP. Risk factors for ITP relapse with vaccination include ongoing treatment, old age [164], and post-splenectomy status or a long history of prior treatment [165]. In terms of platelets, low platelet counts are reportedly associated with a higher risk of relapse [164], and platelet counts often decrease rapidly in patients with normal platelet count [166]. Regardless of the platelet count, attention should be paid to transition of the platelet count in ITP patients. The nadir of platelet counts in ITP patients has been observed around 7–10 days after vaccination [165]. Reports have also recommended measuring platelet counts on days 3–7 after vaccination to confirm the presence or absence of ITP flare-up [167]. Some reports have described ITP cases after vaccination in which the patients were able to receive additional vaccinations without problems [168].

Furthermore, a decrease in platelet count greater than 50% from baseline was observed in 1.0% of healthy controls [164], suggesting that a significant number of patients after vaccination may experience de novo ITP, including cases in which clinical symptoms do not develop and go unnoticed [164].

In patients with paroxysmal nocturnal hemoglobinuria (PNH) or atypical hemolytic uremic syndrome using eculizumab or ravulizumab, vaccination within one week of eculizumab administration and within four weeks of ravulizumab administration is recommended to maintain blood levels of these agents [169]. Thus far, no reports have described an increased risk of relapse of the primary disease after vaccination in specific diseases or a need to refrain from vaccination, and the benefits of vaccination are considered to outweigh the disadvantages. Adequate consultation with the patient and careful observation of changes in coagulation studies are important.

### 3.2. Novel Clotting Abnormalities after Vaccination

Vaccines are being developed and marketed around the world by a number of manufacturers. The number of vaccinations worldwide has exceeded 10 billion, and more than 50% of the global population had completed the required number of vaccinations as of the end of January 2022 [170]. However, the occurrence of thrombosis and bleeding after vaccination remains problematic.

#### 3.2.1. Vaccine-Induced Immune Thrombotic Thrombocytopenia (VITT)

A series of reports have described thrombosis with thrombocytopenia after vaccination with the adenovirus-vectored vaccine (ChAdOx1 nCoV-19) [171,172,173,174]. Similar reports have been made for Ad26.COV2.S, which is an adenovirus vector type vaccine [175,176]. Thrombi occurring after adenovirus-vectored vaccinations have also been characterized as occurring in unusual sites, such as cerebral venous sinuses and visceral veins (e.g., portal vein) [171].

Thrombosis with thrombocytopenia following administration of adenovirus vector vaccines has been referred to as VITT, as well as vaccine-induced prothrombotic immune thrombocytopenia and thrombosis with thrombocytopenia syndrome [177]. VITT is currently the most commonly used term and is considered by the authors as the most appropriate term for describing this condition.

#### 3.2.2. Thrombotic/Bleeding Disorders Other Than VITT

In addition to the adenovirus vector vaccines, ChAdOx1-nCoV-19 and Ad26.COV2.S, the mRNA vaccines, BNT162b2 and mRNA-1273, have been reported to cause various thrombotic/hemorrhagic diseases. Thrombotic/hemorrhagic adverse effects of SARS-CoV-2 vaccines, including those of the adenovirus vector type and the mRNA type, are described below. The diseases that should be differentiated from VITT are shown in Table 3.

(1) Hemorrhagic disease

Complications such as hematuria, extensive petechial hemorrhage, subarachnoid hemorrhage [178], immune thrombocytopenia [179,180,181,182,183], Evans syndrome [184], acquired hemophilia [185], and factor XIII inhibitor [186] have been reported with the use of adenovirus vector and mRNA vaccines. In particular, when platelet transfusion is performed for hemorrhagic disease, examination to rule out VITT should always be performed, because with the presence of VITT in the background, the underlying condition may be aggravated after platelet transfusion.

Since thrombosis after vaccination initially drew worldwide attention, there is a tendency to focus on thrombosis, but it is important to remember that hemorrhagic side effects may also occur.

(2) Thrombotic disease

Reports have described carotid artery thrombosis and aortic thrombotic complications [187], deep vein thrombosis [188,189], myocardial infarction [190], adrenal infarction/hemorrhage [191,192], and cerebral infarction/intracranial hemorrhage [193] following adenovirus vector and mRNA vaccinations. Examination to rule out VITT should always be conducted, not only in the case of hemorrhagic events, but also in the case of thrombotic events, due to the concern that a background of VITT may exacerbate the underlying condition with heparin administration.

(3) Thrombophilia with low platelet count

Numerous reports have described thrombotic thrombocytopenic purpura [194,195,196] and DIC [197]. The exclusion of VITT is still important to determine an accurate treatment strategy.

According to a systematic review of cardiovascular and hematological events after SARS-CoV-2 vaccination, these abnormalities tended to be slightly more frequent among women and young people. Adenovirus-vectored vaccines, ChAdOx1-nCoV-19 and for Ad26.COV2.S, were associated with higher rates of thrombosis and thrombocytopenia, while mRNA vaccines, BNT162b2 and mRNA-1273, were associated with higher rates of cardiac injury in a higher proportion of cases [198]. Although accurate evaluation is difficult due to differences in the number of vaccinations, age group, race, and other factors among manufacturers, certain tendencies in thrombotic/hemorrhagic adverse effects may exist depending on the type of vaccine. Although this remains a subject for further study, knowing which manufacturer’s vaccine a patient received is still important in actual clinical practice.

### 3.3. Pathophysiology of VITT

VITT is characterized by thrombosis occurring at unusual sites such as cerebral venous sinuses and visceral veins (thrombosis in common sites such as venous thromboembolism is also seen), decreased platelet count, and coagulation abnormalities (e.g., elevated D-dimer) occurring 4–28 days after vaccination. In addition, a positive (usually strongly positive) result from enzyme-linked immunosorbent assay (ELISA) for anti-platelet factor 4 (PF4) antibodies is the basis for a definitive diagnosis, even though heparin is not used. As such, the condition of VITT is considered similar to immune (spontaneous) heparin-induced thrombocytopenia (HIT) [199]. An important point is that measurement of anti-PF4 antibodies by chemiluminescent immunoassay (CLIA) or latex agglutination will result in false-negative results, rendering definitive diagnosis impossible with these two methods [177,200]. Importantly, anti-PF4 antibodies should be measured by ELISA. However, false-negative results with the CLIA and latex agglutination methods can also be used as one basis for VITT diagnosis [201].

Although the frequency of VITT is extremely low, ranging from a few to ten cases per million [202], it is important to note that this condition is often fatal when present.

**Figure 3:**
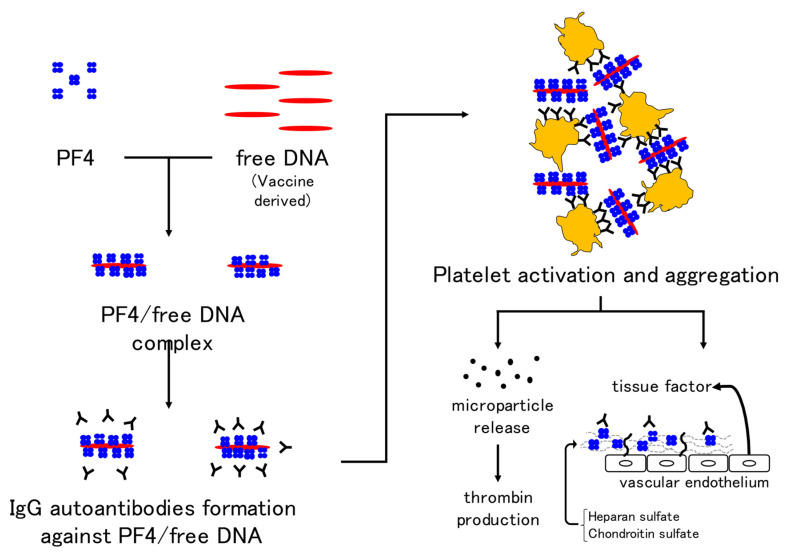
Mechanism of VITT development in adenovirus vector vaccines. Immunoglobulin G autoantibodies against the complex of platelet factor 4 and free DNA in the vaccine result in platelet activation and aggregation. In addition, the release of microparticles and activation of coagulation by vascular endothelial cells are thought to be mechanisms underlying thrombus formation in VITT. Abbreviations: VITT, vaccine-induced immune thrombotic thrombocytopenia; PF4, platelet factor 4.

The mechanism of VITT is thought to be the formation of autoantibodies against the complex of PF4 and free DNA or the coating protein of adenoviruses and binding of the Fc portion of autoantibodies to the Fc receptor on the platelet membrane, which may induce platelet activation and aggregation. In addition, prothrombotic microparticles are released from activated platelets and thrombin production is promoted. PF4 binds to heparin sulfate and chondroitin sulfate on vascular endothelial cells, and binding of autoantibodies to these sites activates endothelial cells. Tissue factor expression from endothelial cells is also thought to activate coagulation [171] (Figure 3). Although 67% (29/43) of healthy controls are positive for anti-PF4 antibodies on day 22 after the first dose of ChAdOx1-nCoV-19, no positive patients showed high antibody titers or developed VITT [203]. When evaluating the anti-PF4 antibody (by ELISA), attention must be paid not only to the positive or negative result, but also to the antibody titer. The prevalence of anti-PF4 antibodies is 1.0–6.6% [204,205] in pre-vaccinated populations. A significant number of post-vaccination anti-PF4 antibody-positive individuals are likely to have had antibodies prior to vaccination, making routine anti-PF4 antibody testing of asymptomatic individuals less useful [206].

Post-vaccination cerebral venous sinus thrombosis has also been reported with BNT162b2 [207,208,209]. However, in all such cases, platelet counts were normal and anti-PF4 antibodies were not detected, therefore, VITT is not a consideration.

The mRNA vaccines work by delivering mRNA (for the formation of spike proteins) protected by lipid nanoparticles to ribosomes in human cells, causing the production of spike proteins [210]. S protein produced by mRNA vaccine is not exactly the same as the S protein of SARS-CoV-2. For example, uridine of mRNA is converted to pseudouridine [211,212] for more efficiency and safety [213]. The mechanism of mRNA vaccine-induced thrombosis may resemble that of COVID-19 [209], although the details are as yet unknown.

### 3.4. Diagnosis of VITT

If a patient presents with any of the symptoms listed in Table 4 within 4–28 days after vaccination (day 0 being the date of vaccination), VITT should be suspected because of the possibility of new thrombosis. Coagulation tests for PT, APTT, fibrinogen, and D-dimer (FDP) are mandatory, and PT and APTT alone are insufficient. Since some VITT patients present with DIC and bleeding tendency, measuring TAT (a marker of coagulation activation) and PIC, plasminogen, and α2PI (as markers of fibrinolysis activation) at the same time is advisable [214].

As mentioned above, measurement of anti-PF4 antibodies by ELISA is of paramount importance, as almost all cases of VITT are positive and show a high titer. However, if VITT is strongly suspected, waiting for the test results may take too long, and initiation of empirical treatment is advisable.

A marked increase in D-dimer to more than four times the upper limit of normal alongside a decreased platelet count is highly suggestive of VITT, but a marked increase in D-dimer is not essential for diagnosis. This is because some VITT patients show only mild elevations in D-dimer.

Another report proposed a “pre-VITT syndrome” in which severe headache at 5–20 days after adenovirus-vectored vaccination should be considered a sign of cerebral venous sinus thrombosis [218]. In fact, although no thrombosis was found on imaging, VITT was suspected early based on clinical symptoms and blood test findings (such as thrombocytopenia and elevated D-dimer), and treatment for VITT was started early, resulting in discharge from hospital in less than one week [219,220]. Notably, VITT is known to occur after administration of adenovirus vector-type COVID-19 vaccines. However, on day 10 after inoculation with the Gardasil 9 vaccine against human papillomavirus (inactivated vaccine), thrombocytopenia similar to that in VITT, venous thrombosis, elevated D-dimer, and the presence of anti-PF4-polyanion complex antibody have been reported [221]. Similar pathologies may have been observed with other vaccinations, but have simply gone unnoticed until now.

Initiating treatment is important when VITT is suspected, as the mortality rate for VITT was an extremely high 50% when the disease was first recognized, although this has now reportedly improved to 22% and even 5% [215]. This is probably due to increased awareness of the disease and earlier detection. The FAPIC score has been devised as a prognostic score looking at fibrinogen, age, platelet count, presence of intracerebral hemorrhage, and presence of cerebral venous sinus thrombosis [222]: fibrinogen < 150 mg/dL; age ≤ 60 years; platelets < 25,000/μL, intracerebral hemorrhage, and cerebral venous sinus thrombosis all represent prognostic factors for poor clinical outcome. This is a simple scoring system, but does not address treatment options for each stratified risk level. In addition, fibrinogen and platelets are suspected to be involved in enhanced-fibrinolytic-type DIC, as described below.

### 3.5. Treatment of VITT (Attention to Fibrinolytic Pathophysiology)

The treatment strategy is based on the treatment of HIT, and includes discontinuation of heparin (although heparin may reportedly be used if HIT can be reliably excluded [215]), high-dose immunoglobulin therapy, anticoagulants other than heparin (argatroban, fondaparinux, DOACs, etc.), and steroids have all been mentioned [223,224].

**Figure 4:**
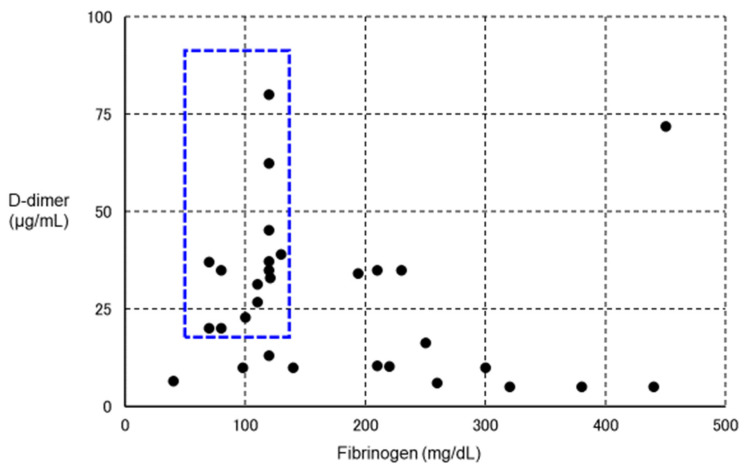
Relationship between VITT and fibrinolytic pathophysiology. We plotted fibrinogen on the horizontal axis and D-dimer on the vertical axis for 30 definitive VITT cases (using data from Reference [222]) in which both fibrinogen and D-dimer values were known. The lower the level of fibrinogen, the higher the level of D-dimer. In particular, patients with blue dotted squares showed a decrease in fibrinogen and a marked increase in D-dimer, suggesting the complication of enhanced-fibrinolytic-type DIC (a subject for further investigation). If anticoagulants are administered to the same intensity as in other cases, the risk of bleeding is considered high. To confirm the diagnosis of enhanced-fibrinolytic-type DIC, measurement of not only the coagulation activation marker TAT, but also the fibrinolysis activation marker PIC is essential. Patients with markedly decreased α_2_PI are at high risk of bleeding. Importantly, in enhanced-fibrinolytic-type DIC, the increase in FDP is more prominent than the increase in D-dimer (discrepancy between FDP and D-dimer). Antithrombin, as a coagulation inhibitor, does not often decrease, even in patients with a marked decrease in α2PI, a fibrinolytic inhibitor (except in patients with reduced hepatic reserve). Abbreviations: VITT, vaccine-induced immune thrombotic thrombocytopenia; DIC, disseminated intravascular coagulation; TAT, thrombin–antithrombin complex; PIC, plasmin–α_2_ plasmin inhibitor complex; α_2_ PI, α_2_ plasmin inhibitor; FDP, fibrin/fibrinogen degradation products.

However, in some VITT patients, in addition to a marked increase in D-dimer, marked decreases in platelet count and fibrinogen have been observed (Figure 4) [171,225], suggesting a change in coagulation markers as in enhanced-fibrinolytic-type DIC [93]. If such patients are reflexively treated with anticoagulant therapy, major bleeding may result, and rigorous evaluation of coagulation and fibrinolytic markers is thus necessary. In fact, a clinical trial of argatroban for DIC was conducted in Japan more than 30 years ago, but was discontinued after the occurrence of bleeding side effects in many patients. In retrospect, we believe that argatroban was more likely to cause bleeding in patients with enhanced-fibrinolytic-type DIC, and should not be prescribed without caution in patients with enhanced-fibrinolytic-type DIC. The characteristics of suppressed-fibrinolytic-type DIC, enhanced-fibrinolytic-type DIC, and VITT are summarized in Table 5.

Tranexamic acid has also been reported to save lives [226], but the use of tranexamic acid in VITT should be considered with care, given the risk of exacerbating thrombosis. Anti-PF4 antibodies persist for at least four months after the end of VITT treatment [227], but advantages [227] and disadvantages [228] are seen with respect to VITT flare-ups, so careful follow-up may be necessary.

In addition, no relapse of VITT occurred after a second additional dose of vaccine was administered to 40 patients who had developed VITT after the first dose. In many cases, mRNA-type vaccines, BNT162b2 and mRNA-1273, had been administered as the second dose, suggesting that immune responses to spike proteins are not involved in the pathogenesis of VITT. From the perspective of protection against infection, a second vaccination should be administered even after the onset of VITT [229].

Cerebral venous sinus thrombosis occurring after vaccination with BNT162b2 (but not after vaccination with ChAdOx1-nCoV-19) did not show a decreased platelet count or anti-PF4 antibodies, was not VITT, and was treated with heparins [208]. Which manufacturer’s vaccine was administered is an important piece of information for determining the course of treatment.

## 4. Summary

The hope is that the spread of novel coronavirus vaccines will bring the COVID-19 pandemic to a close. However, the spread of COVID-19 remains unpredictable. In addition, more careful handling is required for coagulopathies occurring after vaccination.

Appropriate assessment of not only coagulation but also fibrinolysis and fine-tuning of therapy on a case-by-case basis may lead to improved outcomes for COVID-19 and VITT.

## Figures and Tables

**Table 1:** Causes of thrombocytopenia in COVID-19.

1. COVID-19 per se [59,60]
2. Disseminated intravascular coagulation (DIC) [45]
3. Idiopathic thrombocytopenic purpura or immune thrombocytopenia (ITP)
4. Thrombotic thrombocytopenic purpura (TTP)
5. Antiphospholipid antibody syndrome (APS) [79]
6. Hemophagocytic syndrome (HPS) [80,81]
7. Heparin-induced thrombocytopenia (HIT)
8. Drug-induced thrombocytopenia
9. Pseudo-thrombocytopenia

Abbreviation: COVID-19, Coronavirus Disease 2019.

**Table 2:** Causes of bleeding in COVID-19.

1. Side effects of anticoagulation therapy
2. Complications of enhanced-fibrinolytic-type DIC
3. Vascular endotheliitis, fragility of vessel walls
4. Acquired von Willebrand syndrome (during ECMO)
5. Thrombocytopenia
6. Decreased coagulation factors (liver failure, vitamin K deficiency, etc.)
7. Others

Abbreviations: COVID-19, Coronavirus Disease 2019; DIC, disseminated intravascular coagulation; ECMO, extracorporeal membrane oxygenation.

**Table 3:** Diseases that should be differentiated from VITT and their key considerations.

Disease Name	Abbreviation	Important Clinical and Laboratory Findings
Heparin-induced thrombocytopenia	HIT	History of exposure to heparin, 4T’s score
Thrombotic microangiopathy	TMA	Appearance of schizocytes (peripheral blood smear), marked decrease in haptoglobin
Thrombotic thrombocytopenic purpura	TTP	A type of TMA with markedly reduced ADAMTS13 activity with ADAMTS13 inhibitor
Immune thrombocytopenia	ITP	Diagnosis of exclusion. Increased megakaryocytes in bone marrow and positive antiplatelet antibodies assist in diagnosis
Antiphospholipid antibody syndrome	APS	Positive for at least one of the following antibodies: lupus anticoagulant; anticardiolipin antibody; and anti-β_2_ GPI antibody
Paroxysmal nocturnal hemoglobinuria	PNH	Hemolysis (normocytic anemia, elevated reticulocyte, elevated indirect bilirubin, elevated LDH, decreased haptoglobin), presence of PNH type-cells (CD55/59-negative)
Disseminated intravascular coagulation	DIC	PT, APTT, fibrinogen, FDP, D-dimer, AT, TAT, PIC, plasminogen, α_2_PI

Abbreviations: VITT, vaccine-induced immune thrombotic thrombocytopenia; TMA, thrombotic microangiopathy; ADAMTS13, a disintegrin-like and metalloproteinase with thrombospondin type 1 motifs 13; GP, glycoprotein; LDH, lactate dehydrogenase; PT, prothrombin time; APTT, activated partial thromboplastin time; FDP, fibrin/fibrinogen degradation products; AT, antithrombin; TAT, thrombin-antithrombin complex; PIC, plasmin-αplasmin_2_ inhibitor complex; α_2_PI, α_2_ plasmin inhibitor.

**Table 4:** Clinical features indicative of VITT.

Clinical Findings
(1) Onset 4–28 days after vaccination (counting the day of vaccination as day 0)
(2) Symptoms suggestive of stroke (unilateral facial palsy, unilateral motor palsy, language disorder, joint parallax, hemispheric neglect, etc.)
(3) Symptoms suggestive of cerebral venous sinus thrombosis (persistent headache, visual disturbance, seizure, nausea and vomiting, psychiatric symptoms, etc.)
(4) Symptoms suggestive of visceral vein thrombosis (persistent abdominal pain, nausea and vomiting, etc.)
(5) Symptoms suggestive of deep vein thrombosis or pulmonary thromboembolism (pain and swelling in lower limbs, chest and back pain, shortness of breath, etc.)
(6) Hemorrhagic tendencies such as hemorrhagic infarction, petechial hemorrhage, and mottled hemorrhage can also occur.

Abbreviations: VITT, vaccine-induced immune thrombotic thrombocytopenia. (Modified from References [215,216,217]).

**Table 5:** Laboratory findings in typical cases of suppressed/enhanced-fibrinolytic-type DIC and VITT.

Disease		Suppressed-Fibrinolytic-Type DIC	Enhanced-Fibrinolytic-Type DIC	VITT
Underlying disease/cause		Severe sepsis	APL, aortic aneurysm, prostate cancer, etc.	Adenovirus vector type vaccination
Pathophysiology		Activation of coagulation and mild fibrinolysis activation	Activation of coagulation and enhanced fibrinolysis	Antibodies against PF4 are mediated platelet and coagulation activation
Main symptom		Organ damage	Bleeding	Headache, abdominal pain, etc.
Examination findings	Platelet count	Decreased	Decreased	Decreased
	PT	Prolonged	Normal to prolonged	Normal to prolonged *
	APTT	Prolonged	Slightly shortened to prolonged	Normal to prolonged *
	Fibrinogen	Normal to elevated	Decreased	Significantly reduced to normal
	D-dimer	Increased	Increased	Increased
	FDP	Increased	Markedly increased	Increased—markedly increased *
	TAT	Increased	Increased	Increased *
	PIC	Slightly increased	Markedly increased	Increased—markedly increased *
Medical treatment		Anticoagulant therapy	Anticoagulant therapy ± antifibrinolytic therapy	Anticoagulant therapy other than heparin, high-dose immunoglobulin therapy, etc.

* Author’s guess due to insufficient information. Abbreviations: DIC, disseminated intravascular coagulation; VITT, vaccine-induced immune thrombotic thrombocytopenia; APL, acute promyelocytic leukemia; PF4, platelet factor 4; PT, prothrombin time; APTT, activated partial thromboplastin time; FDP, fibrin/fibrinogen degradation products; TAT, thrombin–antithrombin complex; PIC, plasmin–α_2_ plasmin inhibitor complex.
